# Notes on Preparations for the Sick

**Published:** 1888-03

**Authors:** 


### Notes on Preparations for the Sick.

---

**Maltine and Cod Liver Oil. Maltine with Cascara Sagrada.**—The Maltine Manufacturing Company, London. **Liquid Peptonoids with Coca.**—Carnrick & Co., London.

The combination of maltine with twenty-five per cent. of cod liver oil is well known and much appreciated. In addition to the nutritive value of the maltine, derived from malted barley, malted wheat, and malted oats, we have the cod liver oil in a finely divided and easily assimilated form, the whole forming a mixture which can be easily taken either plain or mixed with milk, wine, or water.

The value of cascara sagrada, in small doses three times a day, as a stimulant to peristaltic action is now well recognised; and its bitter taste is disguised by the combination with maltine. Each fluid ounce contains one drachm and a half of the fluid extract of cascara sagrada.

We have previously (March, 1886) given our experience of the Beef Peptonoids in the form of powder, a complete food, containing 95 per cent. of nutritive matter in a palatable form.

The **Liquid Peptonoids** contain the same ingredients, the beef, gluten and milk perfectly digested in a liquid form: it is palatable as a cordial, and must have a high nutritive value.

The combination of this fluid with coca should give us a preparation of the greatest restorative value. The efficacy of coca as a stimulant is generally admitted, but there will be a waste of tissue going on in accordance with the stimulating effect of the drug: it seems reasonable that the combination of the stimulating substance, coca, with the nitrogenous foods in the liquid peptonoids should give us a fluid by means of which stimulation and re-construction may go on simultaneously, the waste being supplied so quickly that no reaction from the stimulants will be present. The depressing effects of the reaction from the use of a substance like coca may go far towards neutralising the benefits derived, and it will clearly act to greater advantage in combination with some nutritive elements ready for assimilation. Such a combination is that devised and prepared by Carnrick and Company, of which each fluid ounce contains the medium dose of powdered beef peptonoids in a soluble condition, combined with thirty grains of coca, the whole forming an agreeable beverage, which must be capable of ready assimilation without trouble to the digestive apparatus.

We have frequently given this fluid, and more particularly for the purpose of counteracting the early morning depression of old people.

It is not altogether a success, inasmuch as some feeling of nausea has frequently followed its administration. The human organism not infrequently fails to appreciate fully all the advantages of the good things provided for it, and we have suspected whether the process of artificial digestion may have been carried a little too far, and that possibly traces of some ptomaine may be present. We hope that some such combination may be found which will be more effectual as a stimulating, easily assimilable, fluid restorative.

---

**Dyspepsia Cakes.**—DAHL'S AGENCY, London.

These so-called "cakes" are said to be "the most excellent and natural remedy for indigestion, dyspepsia and constipation." They contain no drugs, and therefore do not interfere with any other medicinal treatment. It is claimed for them that

"They help the stomach to digest the food thoroughly. They absorb all the burning acid from the stomach. They drive away colic and all its pains effectually. They move the bowels regularly and without trouble. They also remove piles."

A food which performs the above stated functions must be invaluable to suffering humanity. They are to be taken soaked in tea or any other fluid, one cake at breakfast and one at supper; and no alteration need be made in the ordinary diet. We doubt not the efficacy of this method, but unfortunately the invalid is not always able to take what is best for him: these cakes would serve admirably as food for the ostrich or the emu, but the stomach of the delicate invalid will probably positively refuse to admit them.

The brown or bran bread used to counteract habitual constipation is commonly found to be more difficult of digestion than the finer kinds of bread: these cakes, made from the "sacks and covers of several kinds of grain," are coarser than the coarsest bran bread we have met with, and are likely to be correspondingly efficient as an irritant to an atonic sluggish mucous membrane needing powerful stimulation. We are inclined to think that organisms destitute of a gizzard will extract little nutriment from these cakes; but if they will stimulate the mucous membrane to active secretion and stimulate the muscular walls to increased activity, they will enable an enfeebled digestive apparatus to utilise more thoroughly the ordinary food supplied. The experience of the individual can alone show whether the coarse compound called "cake" will perform what is promised for it.

---

**Essence of Beef. Pure Beef Tea. Real Turtle Soup.**—The Viking Food & Essence Co., Limited, London.

These preparations, mounted in glass bottles, are prepared from the best materials and put up with every possible care. They are moderate in price, and valuable to most invalids who are not able to digest ordinary solid foods.

The **Essence** may be taken cold as jelly, as also may the essences of chicken, turtle, mutton and veal.

The **Concentrated Beef Tea** is ready for use; less than one ounce will make a cup of excellent beef tea in a few minutes. It is also suitable for addition to soups, gravies, &c.

The **Invalid Turtle Soup** in quarter pints, just enough for one, seems likely to be often appreciated.

---

**Sanitary Rose Powder. Phenate of Soda Solution.**—James Woolley, Sons & Co., Manchester.

The well-known powder, pink or white, has antiseptic, deodorant and mildly astringent properties, which render it specially serviceable as a dusting powder for infants and for aged or infirm people troubled with incontinence. It has the advantage of being soluble in water, and does not cake upon the skin, or leave any concretion to excite irritation. It is also useful for fœtid perspiration, as also for tender feet. Those who walk much will find comfort from a little of the powder dusted inside the sock or stocking.

The solution is a powerful antiseptic and disinfectant, but without the strongly caustic properties of carbolic acid.

As a mouth wash diluted with twenty parts of water, and to allay cutaneous irritation diluted with twelve parts of water, and for chilblains with twice its bulk of water, it has been found to be very efficient.

---

**Jensen's Ice Refined Cod Liver Oil.**—HERTZ & COLLINGWOOD, London.

To the numerous and increasing body of invalids for whom the various forms of cod liver oil are a necessity, the gradual improvement in the appearance and taste which has gone on for many years has been a real advantage. The old strongly flavoured and dark-coloured varieties, unpleasing to the eye as to the palate, have been replaced by the newer brands, perfectly pure, light in colour, and with a sweet, not unpleasant flavour. Of these newer brands, we have not seen a more attractive one than that prepared by the Jensen Company at Brettesnoes, Lofoten Islands, Norway, the seat of the great Norwegian cod fisheries; it is obtained from quite fresh livers, so that it is free from rancid odour and nauseous flavour, being almost odourless and tasteless.

---

**Pure Concentrated Cocoa. Malted Cocoa.**—J. S. FRY & SONS, Bristol.

The **Concentrated Cocoa** has the special characteristic of extreme solubility, and is therefore of value to those of weak digestion. It is prepared by a new process, which includes thorough cleaning and cooking of the bean, and culminates in the conversion of its substance into soluble starch and dextrine. Chemical tests show that the bean has been completely broken up, the cellulose completely removed, and the amylaceous elements rendered soluble and easily digestible. Its flavour, solubility, purity, and nutritive value must give it a high position in the list of foods for the sick. The **Malted Cocoa** is a combination of cocoa extract with concentrated extract of malt. Its dietetic and digestive value is beyond question, more especially to those who require artificial digestive aid. It has been deprived of superfluous oil, and, being malted, does not thicken in the cup.

---

**Geddes's Fluid Extract of Hemlock Bark.**—THE GEDDES MANUFACTURING CO., London.

This fluid is a highly concentrated extract from the bark of the common Canadian hemlock tree; it is reduced by evaporation to the consistency of thick syrup, is soluble in water, contains no alcohol, will not ferment, and will keep in any climate.

It is said to be of great value for the treatment of chronic sores, for stopping the flow of blood, and as an application to the uterine mucous membrane in chronic leucorrhœa from endometritis. It appears to act as a tonic astringent, its application is painless, and it is spoken of in the highest terms by those who have had much experience of its action.

---

**Pharmaceutical Preparations.**—T. Buxton, Bristol.

Mr. Buxton has sent us several of his preparations.

**Medicated Gelatine Applications.**—These consist of prepared gelatine, into which are incorporated various medicinal substances to be used as applications to the skin, instead of ointments or plasters, than which they are more cleanly, whilst equally effectual. The following are in use:

Chrysophanic Gelatine.
Carbolic ,,
Iodine ,,
Iodoform ,,
Morphia ,,
Zinc oxide ,,
Zinc oleate ,,
Belladonna ,,

They are liquefied by placing the bottle in hot water, and may then be painted on the part with a brush.

**Tonic Coca Pastilles.**—These contain a concentrated essence (prepared from the fresh green leaves) of the well-known coca of Peru; each contains a quantity equivalent to an ordinary dose of coca wine; they have the aroma of the coca plant, and they produce the physiological effects of coca on the mucous membrane of the mouth. The pastille is a portable and convenient form for administration, and we have found that one or two of them do produce an appreciable effect on the nervous system in warding off fatigue and exhaustion.

**Liquor Cascaræ Aromaticus** is an agreeable form in which the nauseous cascara sagrada may be given. It is a palatable and efficient preparation, in doses of one to two fluid drachms twice daily.

**Pil. Pepsinæ.**—One pill is guaranteed to dissolve one thousand grains of coagulated albumen, by the aid of hydrochloric acid at a temperature of 100°, in six hours. A pill such as this, coated and tasteless, obviously presents advantages over both the nauseous pepsine powders and the liquids which are superseding them.

**Liq. Cinchonæ Aromaticus.**—One drachm contains, in an agreeable form and readily miscible with water, an equivalent of ten grains of the powdered bark.

**Liq. Saccharini.**—This fluid, containing five per cent of saccharine, five minims being equal to a drachm of simple syrup, is of great convenience in dispensing, and more especially where sugar is undesirable either from the presence of diabetes or a tendency to adiposity.

---

**"The Leicester" Adhesive Plaster.**—A. de St. Dalmas, Leicester.

We have received a specimen of this adhesive plaster. For some time we have used plaster of this sort for every purpose in surgery where adhesive plaster may be used, and we consider it the best in the market, not only in respect of its adhesive qualities, but also on account of its strength and durability. In price it is most reasonable.

---

Mr. Alfred Carter sends us his illustrated and descriptive catalogue of every description of mechanical appliance for the aid and comfort of the sick and feeble.

A great variety of invalid's chairs and couches, bed tables, reading machines, spinal carriages, bed lifts, back rests, leg rests, stretchers, walking machines, crutches, and many other useful articles are described and figured. We notice the patent "pillow divider:" an apparatus which might be effectual in preventing the contagion of phthisis from being conveyed to husband or wife.

---

With reference to a criticism which we made in the last number of the *Journal* on Milne's sal. alembroth antiseptic dressings, that they are liable to cause pustular eruptions on the skin if left undisturbed for a week or longer, Mr. Milne makes the following statement:

"The Editor refers to the one fault of the alembroth dressings. The sublimate, being readily soluble in the discharges, is washed through the layers to the edges, where it is deposited in excessive quantities; hence the eruptions. On this account, absorbent gauze and cotton wool prepared with bin-iodide of mercury are being substituted in special cases."

